# Supplementary material for: GPC1 specific CAR-T cells eradicate established solid tumor without adverse effects and synergize with anti-PD-1 Ab
Source: eLife. 2020 Mar 31;9:e49392. doi: 10.7554/eLife.49392 (PMC7108862; doi:10.7554/eLife.49392)
Supplement: Figure 4—figure supplement 1—source data 1. [file elife-49392-fig4-figsupp1-data1.docx]

**Figure 4—figure supplement 1.** The sequences of GPC1-specific murine CAR vectors.

mCAR

ATGGCCTCACCGTTGACCCGCTTTCTGTCGCTGAACCTGCTGCTGCTGGGTGAGTCGATTATCCTGGGGAGTGGAGAAGCTGCGGTGACGTTGGACGAGTCCGGGGGCGGCCTCCAGACGCCCGGAAGAGCGCTCAGCCTCGTCTGTAAGGCCTCCGGGTTCACCTTCAGCCGTTACGCCATGTACTGGGTGCGACAGGCGCCCGGCAAGGGGCTGGAGTTCGTCGCTGGTATTGGCAACACTGGTAGATACACAGGCTACGGGTCGGCGGTGAAGGGCCGTGCCACCATCTCGAGGGACAGCGGGCAGAGCACAGTGAGGCTGCAACTGAACAACCTCAGGGCTGAGGACACCGGCAACTACTACTGCGCCAAAAGTGTTAGTCCTTACTGTTGTGATGCTGCTGACATCGACGCATGGGGCCACGGGACCGAAGTCATCGTCTCCTCCGGCGGTGGCGGATCAGGTGGCGGTGGAAGTGGCGGTGGTGGGTCTGCTAGCACTCAGCCGTCCTCGGTGTCAGCAAACCCAGGAGAAACCGTCAAGATCACCTGCTCCGGGGGTAGCAGTGGCTATGCTTATGGCTGGTACCAGCAGAAGTCTCCTGGCAGTGCCCCTGTCACTCTGCTCTATAGCAACAACAACAGACCCTCGGACATCCCTTCACGATTCTCCGGTTCCAAATCCGGCTCCACAGCCACATTAACCATCACTGGGGTCCAAGCCGAGGACGAGGCTGTCTATTTCTGTGGGAGTGTAGACAGCAGCAGTTATGCTGGTATATTTGGGGCCGGGACAACCCTGACCGTCCTAGCGGCCGCAATTGAGTTCATGTACCCTCCGCCTTACCTAGACAACGAGAGGAGCAATGGAACTATTATTCACATAAAAGAGAAACATCTTTGTCATACTCAGTCATCTCCTAAGCTGTTTTGGGCACTGGTCGTGGTTGCTGGAGTCCTGTTTTGTTATGGCTTGCTAGTGACAGTGGCTCTTTGTGTTATCTGGACAAATAGTAGAAGGAACAGACTCCTTCAAAGTGACTACATGAACATGACTCCCCGGAGGCCTGGGCTCACTCGAAAGCCTTACCAGCCCTACGCCCCTGCCAGAGACTTTGCAGCGTACCGCCCCAGAGCAAAATTCAGCAGGAGTGCAGAGACTGCTGCCAACCTGCAGGACCCCAACCAGCTCTACAATGAGCTCAATCTAGGGCGAAGAGAGGAATATGACGTCTTGGAGAAGAAGCGGGCTCGGGATCCAGAGATGGGAGGCAAACAGCAGAGGAGGAGGAACCCCCAGGAAGGCGTATACAATGCACTGCAGAAAGACAAGATGGCAGAAGCCTACAGTGAGATCGGCACAAAAGGCGAGAGGCGGAGAGGCAAGGGGCACGATGGCCTTTACCAGGGTCTCAGCACTGCCACCAAGGACACCTATGATGCCCTGCATATGCAGACCCTGGCCCCTCGCTAA
